# Widespread Pyrethroid and DDT Resistance in the Major Malaria Vector *Anopheles funestus* in East Africa Is Driven by Metabolic Resistance Mechanisms

**DOI:** 10.1371/journal.pone.0110058

**Published:** 2014-10-15

**Authors:** Charles Mulamba, Jacob M. Riveron, Sulaiman S. Ibrahim, Helen Irving, Kayla G. Barnes, Louis G. Mukwaya, Josephine Birungi, Charles S. Wondji

**Affiliations:** 1 Vector Biology Department, Liverpool School of Tropical Medicine, Pembroke Place, Liverpool, United Kingdom; 2 Uganda Virus Research Institute, Entebbe, Uganda; National Institute for Communicable Diseases/NHLS, South Africa

## Abstract

**Background:**

Establishing the extent, geographical distribution and mechanisms of insecticide resistance in malaria vectors is a prerequisite for resistance management. Here, we report a widespread distribution of insecticide resistance in the major malaria vector *An. funestus* across Uganda and western Kenya under the control of metabolic resistance mechanisms.

**Methodology/Principal Findings:**

Female *An. funestus* collected throughout Uganda and western Kenya exhibited a *Plasmodium* infection rate between 4.2 to 10.4%. Widespread resistance against both type I (permethrin) and II (deltamethrin) pyrethroids and DDT was observed across Uganda and western Kenya. All populations remain highly susceptible to carbamate, organophosphate and dieldrin insecticides. Knockdown resistance plays no role in the pyrethroid and DDT resistance as no *kdr* mutation associated with resistance was detected despite the presence of a F1021C replacement. Additionally, no signature of selection was observed on the sodium channel gene. Synergist assays and qRT-PCR indicated that metabolic resistance plays a major role notably through elevated expression of cytochrome P450s. DDT resistance mechanisms differ from West Africa as the L119F-GSTe2 mutation only explains a small proportion of the genetic variance to DDT resistance.

**Conclusion:**

The extensive distribution of pyrethroid and DDT resistance in East African *An. funestus* populations represents a challenge to the control of this vector. However, the observed carbamate and organophosphate susceptibility offers alternative solutions for resistance management.

## Background

Malaria burden remains high in sub-Saharan Africa where 90% of the 630,000 malaria deaths occurs [1]. Uganda is one of the four African countries which accounted for ∼50% of deaths due to malaria reported on the African continent in 2010, the other three countries being Nigeria, Democratic Republic of Congo, and Ethiopia [2].

Malaria is hyperendemic in Uganda and is the main cause of morbidity and mortality with about 100,000 deaths recorded annually (Ugandan Ministry of Health). About 50% of the reported fatalities occur in under-five children and nearly a third of the deaths are reported in pregnant women. Malaria control in Uganda relies heavily on vector control through the use of long-lasting insecticide nets (LLINs) and indoor residual spraying (IRS) [1]. However, resistance to the main insecticides in the major malaria vectors such as *An. gambiae* and *An. funestus* is threatening the success of these control interventions.

Resistance to different classes of insecticides used in public health is increasingly reported in *An. funestus* prompting fears that it could disrupt control programs against this vector and malaria control in general. Indeed, resistance to pyrethroids, DDT and carbamates has been detected in different regions of Africa including southern [3]–[6], Central Africa [7] and West Africa [8], [9]. In Uganda, pyrethroid resistance until recently was mainly reported in *An. gambiae* [10] while *An. funestus* was considered to be susceptible to most insecticides and therefore not a concern for control program interventions. The detection of pyrethroid and DDT resistance in *An. funestus* populations from Tororo district in the eastern region of Uganda in 2010 [11] changed this view and prompted fears that this resistance could spread rapidly throughout Uganda and neighbouring countries and disrupt malaria control as previously seen in southern Africa [12], [13]. The *An. funestus* population from Tororo was found to be resistant to both type I (permethrin) and type II (deltamethrin) pyrethroids but contrary to populations from southern Africa, was resistant to DDT [7], [11].

The detection of such resistance to pyrethroids and DDT in a location close to the border with Kenya suggests that resistance distribution could extend to other districts in Uganda and also in Kenya. However, there is currently no knowledge about the extent of this resistance and its geographical distribution. Consequently, it remains unknown whether the same control strategy could efficiently control this vector throughout Uganda and the neighbouring countries. Furthermore, if resistance is present throughout Uganda, it remains unknown whether it is driven by the same underlying resistance mechanisms or not. Such information is essential in designing suitable control interventions across the region and to improve the implementation of resistance management strategies against *An. funestus* as recommended by the WHO Global Plan for Insecticide Resistance Management (GPIRM) [14].

To fill these knowledge gaps, we report here a nationwide resistance profiling of six Ugandan populations of *An. funestus* and one population from western Kenya revealing a widespread distribution of pyrethroid and DDT resistance. The role of metabolic and the knockdown resistance (*kdr*) mechanisms are established showing that resistance mechanisms in East Africa significantly differ to that observed in southern or West Africa. In addition, the contribution of *An. funestus* in the malaria transmission was also assessed.

## Methods

### Study sites

**Figure 1:**
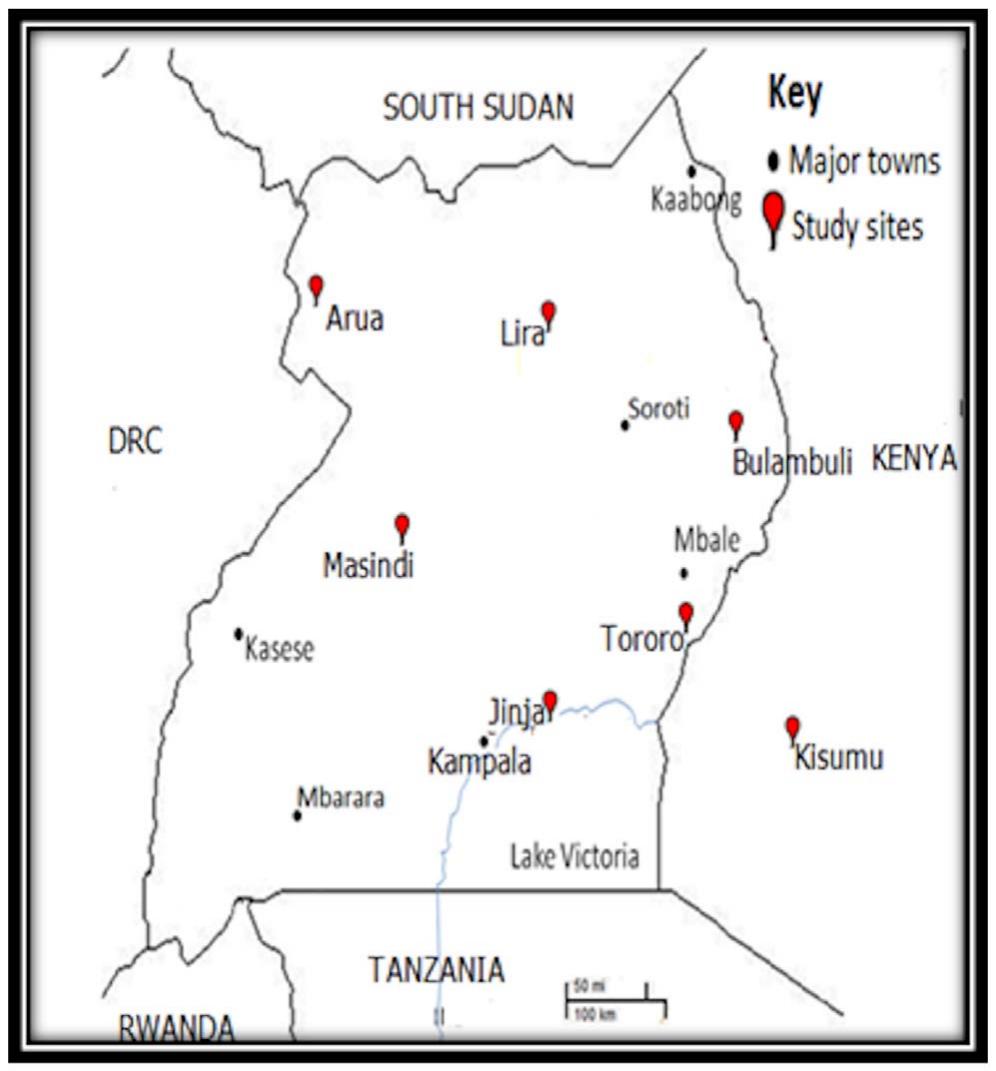
Map of Uganda showing study districts.

Adult Anopheles mosquitoes were collected from six districts in Uganda; Bulambuli (Bl) in North-East (1°10′N, 34°23′E), Lira (Lr) in North central (2°14′N, 32°54′E), Masindi (Ms) in West-central (1°40′N, 31°42′E), Jinja (Jn) in South central (0°26′N, 33°12′E), Arua (Ar) in North West (3°1′N, 30°54′E) and Tororo (Tr) in East (0°41′N, 34°10′E). A similar collection was made from Kisumu-Siaya (Ks) district (0°05′S, 34°15′E) in West Kenya (Figure 1). Sample villages in these districts are located in close proximity with rivers, swamps and tributaries joining major rivers as these permanent water bodies are suitable breeding sites for *An. funestus*. The surrounding vegetation was mainly shrubs, maize crops, ‘finger’-millet, banana and coffee plantations. Subsistence farming is the main practice with exception of Tororo and Kisumu–Siaya where large scale commercial rice farming is practiced as well. Housing facilities were mostly traditional homes dominated by grass thatched and semi-permanent iron roofed dwellings. No specific permissions were required for these locations/activities and these field collections did not involve endangered or protected species.

### Mosquito collections

Indoor resting, blood fed female adult *Anopheles* mosquitoes were collected from houses between 06.00 a.m. and 12.00 p.m., following verbal consent from village Local Council 1 (L.C1) chairpersons and household owners. No specific permissions were required for these locations/activities and these field collections did not involve endangered or protected species. Mosquito collections were carried out between December 2011 and June 2012, between end of dry season and beginning of the rainy season, with temperatures and relative humidity ranging from 26°C to 29°C and 66% to 77% respectively. Mosquitoes were collected into paper cups with netted lids using manual aspirators, kept in a cooling box and immediately transported to the insectary at the Uganda Virus Research Institute based in Entebbe, Uganda.

A forced-egg laying method was used to induce the females to lay eggs [11]. Eggs were stored at room temperature for up to 3 days and were transferred to the Liverpool School of Tropical Medicine (LSTM), UK (under the LSTM import license from DEFRA). The egg batches were hatched in small paper cups and transferred to plastic larvae bowls for rearing as previously described by [4], [11].

### Species Identification

Field caught females (F_0_s) which oviposited first generation (F_1_) egg batches were morphologically identified as belonging to the *An. funestus* group according to the key of [15]. Genomic DNA was extracted from head and thorax using the Livak protocol described in [16]. A cocktail PCR described by [17] was performed to identify member species of the *An. funestus* group.

### Estimation of the sporozoite infection rates

The sporozoite infection rate was determined using a TaqMan assay [18]. The real-time PCR MX 3005 (Agilent, Santa) system was used for the amplification according to the protocol previously described by Bass et al [18]. Primers [18] were used together with two probes labelled with fluorophores, FAM to detect *Plasmodium falciparum*, and HEX to detect *P. ovale, P. vivax and P. malariae* [18]. Two *P. falciparum samples* and a mix of *P. ovale, P. vivax* and *P. malariae* were used as positive controls.

A nested PCR was performed for all the positive samples to validate the TaqMan assay as described by [19].

### Adult mosquito susceptibility assays

Following WHO procedures [20], F_1_ adults aged between 2–5 days were exposed for 1 hour to insecticide impregnated papers. Nine insecticides belonging to the four major public health classes of insecticide were variously tested: the pyrethroids permethrin (0.75%), deltamethrin (0.05%), lambda-cyhalothrin (0.05%) and etofenprox (0.05%); the organochlorines DDT (4%) and dieldrin (4%); the carbamate bendiocarb (0.1%); and the organophosphates fenitrothion (5%) and malathion (5%). Each test included control mosquitoes exposed to untreated papers.

### PBO synergist assays

F_1_ adults were pre-exposed to pyperonyl butoxide (PBO) (4%) paper for 1 hour and immediately exposed to 0.75% permethrin and 4% DDT for another 1 hour. Mortality after 24hrs was assessed and compared to the results obtained without PBO.

### Polymorphism analysis of the voltage-gated sodium channel (VGSC) gene

A fragment of the VGSC gene spanning a portion of intron 19 and the entire exon 20 including the 1014 codon associated with knockdown resistance (kdr) in *An. gambiae* [21], [22] was amplified and sequenced in pyrethroid/DDT resistant and susceptible mosquitoes from Tororo, Balambuli, Lira, Jinja and Kisumu in order to detect possible mutations associated with pyrethroid/DDT resistance and/or to assess a possible correlation between polymorphism patterns of this gene and resistance phenotypes. Genomic DNA was extracted using the LIVAK method [16] and amplified using primers listed in Table S1. The PCR was carried out as previously described [11]. PCR products were cleaned, sequenced and then aligned using ClustalW [23] while haplotypes reconstruction and polymorphism analysis were done using DnaSP v5.10 [24]. All DNA sequences have been submitted to Genbank (Accession Number: KM037758 - KM037915).

#### Genetic variability of the VGSC gene across Ugandan and Kenyan populations

The patterns of genetic variability and differentiation of the *VGSC* gene across Ugandan populations of *An. funestus* and a western Kenyan population in relation to both permethrin and DDT resistance was assessed by estimating the levels of pair-wise genetic differentiation between the populations as implemented in dnaSP 5.10 using the *K_ST_* statistic [25]. The significance of the *K_ST_* estimates was assessed by permutation of subpopulation identities and re-calculating *K_ST_* 10,000 times.

#### Phylogenetic tree of *VGSC* haplotypes

A maximum likelihood phylogenetic tree was constructed for the VGSC haplotypes across Uganda and for the Kisumu Kenyan samples using MEGA 5.2 [26]. The best-fit substitution model was firstly assessed based on the Bayesian Information Criterion (BIC) and used to generate the maximum likelihood tree as implemented in MEGA 5.2 with 500 bootstrap replications to assess the robustness of the tree.

### Transcription profiling of candidate metabolic resistance genes

The expression profile of candidate resistance genes previously associated with either pyrethroid (*CYP6P9a and CYP6P9b*) or DDT (*GSTe2*) resistance in *An. funestus* populations in other African regions [5], [27] was assessed using a quantitative Reverse Transcriptase PCR (qRT-PCR). Total RNA was extracted from each sample set which included: three batches of 10 F_1_ resistant female mosquitoes (R1–R3) (2–5 days old) that survived after 1 h exposure to permethrin or DDT in the different locations; and 3 batches of 10 female mosquitoes from the fully susceptible *An. funestus s.s* strain FANG (S1–S3). The protocol used for RNA extraction, cDNA synthesis and qRT-PCR reactions has been previously reported [5]. Expression and fold change of each gene in resistant (R) and FANG susceptible (S) were calculated according to 2^-ΔΔCT^ method [28] following normalization with housekeeping genes RSP7 ribosomal protein S7 (AGAP010592) and the Actin 5C (AGAP000651) genes [5].

### Role of the L119F-GSTe2 mutation in the DDT resistance in Uganda

The L119F-GSTe2 mutation was recently shown to play a major role in the DDT resistance in West Africa [27]. To assess the role of this mutation in the DDT resistance in Uganda a Taqman assay [27], was used to genotype 30 mosquitoes dead after 1 h exposure to 4% DDT (susceptible) and 30 mosquitoes alive (resistant) from Jinja. The TaqMan reactions were performed as previously described [27] in a 10- µl final volume containing 1×SensiMix (Bioline), 800 nM of each primer and 200 nM of each probe using the Agilent MX3005P machine.

## Results

### Mosquito field collections and rearing

A total of 2048 F_0_s were collected from across the seven study districts combined. The highest number were collected from Bulambuli (n = 391) and the least number from Arua (n = 201). Results from PCR-species identification confirmed that the F_0_ adult females that oviposited F_1_ egg batches were all *An. funestus* in all locations apart from Masindi where a mixed population of *An. funestus s.s.* and *An. parensis* were observed [29]. Other *Anopheles* species mainly from *An. gambiae* complex were also found in the same study areas but no attempts were made to obtain egg batches from non-members of the *An. funestus* group. Over 7000 F_1_ adults were generated from total of 683 F_1_ egg batches at the time of rearing.

### Sporozoite infection rates

A total of 18 out of 286 field-caught *An. funestus* were positive for *Plasmodium falciparum* sporozoites (Table S2). *Plasmodium falciparum* was the only detected malaria species in all locations with Lira having the highest infection rate (10.4%) whereas both Masindi and Arua had the lowest (4.2%).

### Susceptibility profiles to insecticides

A total of 5,057 F_1_ adult mosquitoes were tested for susceptibility following 1 hour exposure to nine insecticides (Table S3).

### Pyrethroids resistance

**Figure 2:**
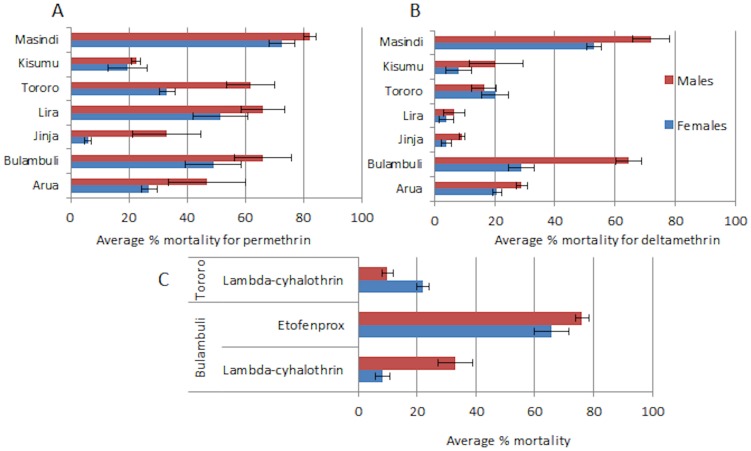
Resistance profiles for pyrethroids in *An. funestus* populations Uganda and Kenya (Kisumu). (A) is for permethrin and (B) for deltamethrin.

A widespread resistance was observed for females and males against both type I and type II pyrethroids throughout the six locations in Uganda and in the Kenyan population from Kisumu (Table S3; Figure 2A). The highest resistance level to permethrin (type I pyrethroids) was observed in Jinja (6±1.2% mortality) and the lowest level in Masindi (over 70±4.3% mortality).

High levels of resistance were also observed against deltamethrin (type II pyrethroid) in all locations with mortality rate in females below 30±4.3% except Masindi where a mixture of *An. funestus* and *An. parensis* was reported. The highest deltamethrin resistance was observed in Lira and Jinja populations with only 4±2.6% mortality (Table S3; Figure 2B).

High resistance was also observed against other pyrethroids (Table S3; Figure 2C) notably against lambda-cyhalothrin in Bulambuli (8±2.4% mortality) and Tororo (22±2.4%). Similarly, resistance was observed against the pseudo-pyrethroid etofenprox in these two locations (66±5.7%).

### DDT resistance

**Figure 3:**
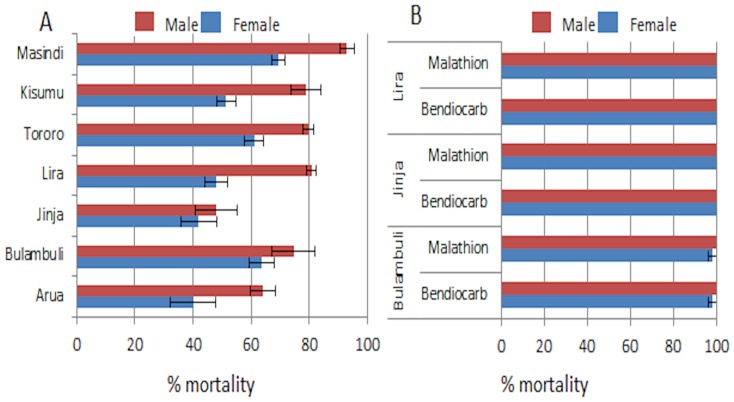
Resistance profiles for DDT (A) and other insecticides (B) in *An. funestus* populations Uganda and Kenya.

A widespread resistance against DDT was observed in all Ugandan locations and in Kisumu (Kenya) with the lowest mortality rate observed in Arua and Jinja (40±7.8 and 42±6.2% mortality respectively) (Table S3; Figure 3A).

### Other insecticides

Despite a small sample size in some cases, bioassays revealed that most *An. funestus* populations were fully susceptible to organophospates (malathion and fenitrothion) and carbamates (bendiocarb) with ∼100% mortality rate. However, slightly reduced mortality rates were observed against bendiocarb in Tororo (96±0%) and Balambuli (98±2.0%), and against malathion in Balambuli (98±2.0%). Test with dieldrin indicated a full susceptibility in Balambuli (Table S3; Figure 3B).

### Synergist assay with PBO

**Figure 4:**
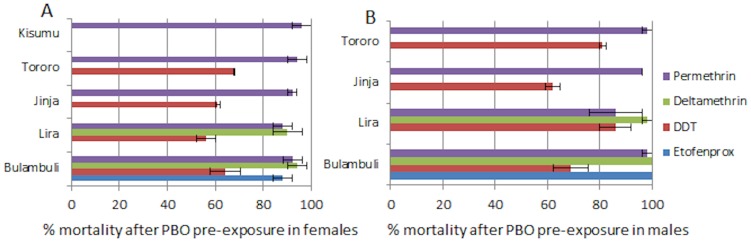
Susceptibility profile after synergist assay with PBO.

Significant recovery of susceptibility to pyrethroids was observed after 1 h pre-exposure to PBO with mortality of over 92±4% observed for permethrin and deltamethrin in Bulambuli, Tororo, Jinja and Kisumu (Figure 4). However, no such recovery of susceptibility was observed for DDT as there were no significant differences in DDT mortality after pre-exposure to PBO.

### Analysis of the polymorphism of the VGSC gene to detect potential kdr mutations

#### Polymorphism analysis and mutation detection

A 917 bp portion of the VGSC gene spanning intron 19 and the entire exon 20 (207 bp) was successfully sequenced in a total of 79 resistant and susceptible mosquitoes from four locations in Uganda (Balambuli, Tororo, Jinja and Lira) and from Kisumu in Kenya (Table 1). Overall, high polymorphism was observed for both resistant and susceptible samples for permethrin and DDT in all locations with a total of 54 substitutions detected (Table 1). A single amino acid change was detected at codon 1021 leading to replacement of phenylalanine with cysteine. Surprisingly, this mutation was observed only in two DDT susceptible mosquitoes from Lira.

**Table 1:** Summary statistics for polymorphism at the sodium channel gene in susceptible and resistant *An. funestus* in Uganda and Kenya.

Samples	N	S	π (k)	h(hd)	Syn	Nonsyn	D	D*
**Permethrin**								
**All resistant**	64	26	0.0041(3.81)	37(0.958)	1	0	−0.97^ns^	−0.13^ns^
**All susceptible**	44	27	0.0042(3.81)	30(0.975)	0	1(F1021C)	−1.29^ns^	−2.13^ns^
**Total**	108	40	0.0042(3.85)	61(0.969)	1	1(F1021C)	−1.53^ns^	−1.99^ns^
**DDT**								
**All resistant**	26	23	0.0052(4.78)	19(0.972)	1	0	−0.75^ns^	0.59^ns^
**All susceptible**	24	25	0.0044(4.08)	21(0.989)	0	0	−1.45^ns^	−1.42^ns^
**Total DDT**	50	33	0.0049(4.5)	34(0.980)	1	0	−1.35^ns^	−0.06^ns^
**Per location**								
**Balambuli**	28	19	0.004(3.7)	19(0.958)	1	0	−0.84^ns^	−0.93^ns^
**Jinja**	30	21	0.0049(4.5)	17(0.959)	1	0	−.063^ns^	0.5^ns^
**Kisumu**	32	23	0.0041(3.8)	24(0.974)	0	0	−1.15^ns^	−1.16^ns^
**Lira**	30	24	0.0043(3.98)	23(0.982)	0	1(F1021C)	−1.21^ns^	−0.46^ns^
**Tororo**	38	21	0.0038(3.45)	21(0.929)	0	0	−1.03^ns^	−1.58^ns^
**Overall Total**	158	54	0.0044(4.11)	82(0.978)	2	1(F1021C)	−1.77*	−1.70^ns^

N, number of sequences (2n); S, number of polymorphic sites; h, Number of haplotypes (haplotype diversity); π, nucleotide diversity (k =  mean number of nucleotide differences); D and D*, Tajima's and Fu and Li's statistics; ns, not significant.

#### Correlation between resistance phenotype and haplotype diversity

**Figure 5:**
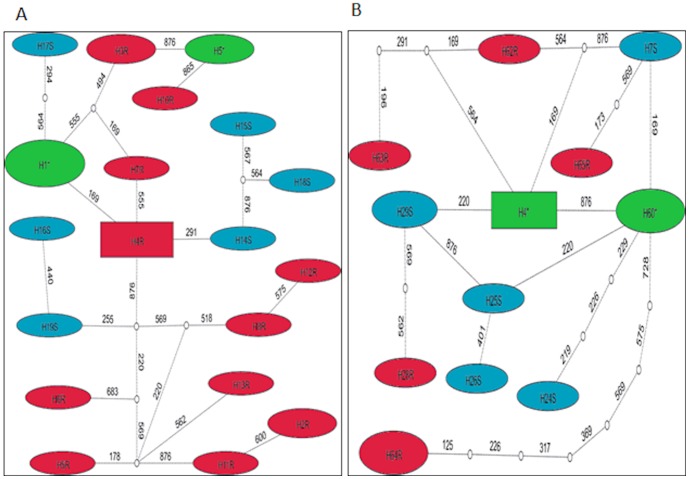
TCS network for the VGSC haplotypes between susceptible and resistant permethrin samples in Bulambuli (A) whereas (B) is for DDT samples in Jinja; Haplotypes are represented as an oval or a rectangle shape, scaled to reflect their frequencies. Lines connecting haplotypes and each node represent a single mutation event (respective polymorphic positions are given above branches). Blue shapes represent haplotypes unique in susceptible mosquitoes; Green shapes represent haplotypes commonly found in resistant mosquitoes and dead mosquitoes; Red shapes represent haplotypes unique to resistant mosquitoes.

Analysis of the maximum likelihood phylogenetic tree of the VGSC sequences did not reveal an association between VGSC polymorphism and pyrethroid (Figure S1A) or DDT (Figure S1B) resistance as no haplotype cluster was associated with a specific resistance haplotype. This trend was confirmed when all samples were combined (Figure S2). This lack of correlation was further supported by the TCS haplotype networks which also did not show a clustering of haplotypes according to their resistance phenotype. In addition the relatively high polymorphism of the VGSC is shown by the high number of haplotypes and the absence of a predominant haplotypes both for permethrin samples in Balambuli (Figure 5A) and for DDT samples in Jinja (Figure 5B). This suggests that the VGSC is probably evolving neutrally contrary to what is expected if knockdown resistance was playing a significant role in the observed pyrethroid and DDT resistance. This absence of selection is further supported by estimates of Tajima D and Fu and Li D* which are all non-significant across all locations.

#### Genetic differentiation

Low to moderate levels of differentiation are observed between the five populations. The lowest and non-significant *K_ST_* of 0.0076 is observed between the Eastern-Ugandan population of Balambuli and that of Kisumu in Kenya while the highest *K_ST_* of 0.078 is observed between Tororo (East central) and Lira in the North. This overall low level of genetic differentiation is in accordance with the lack of haplotype clustering according to geographical location observed in the maximum likelihood phylogenetic tree.

### Transcription profiling of candidate metabolic resistance genes

**Figure 6:**
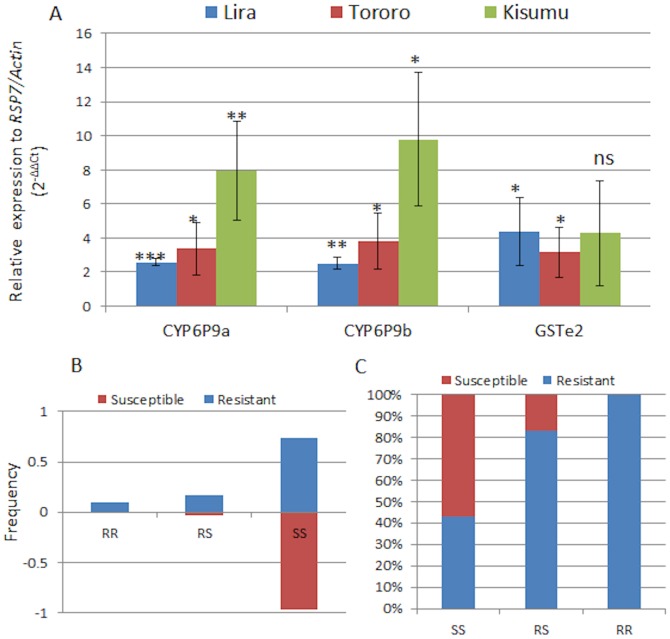
Investigation of metabolic resistance mechanisms. (A) Differential expression of candidate resistance genes by qRT-PCR between resistant mosquitoes (Lira, Tororo and Kisumu) and the susceptible FANG strain; Error bars represent standard deviation (n = 3). *** P<0.001; ** P<0.01; *P<0.05; ns not significant. (B) Correlation between the L119F alleles and DDT resistance phenotype. (C) Genotype distribution of L119F mutation between DDT resistant and susceptible mosquitoes.

A significant over-expression of the cytochrome P450s *CYP6P9a* and *CYP6P9b* known to confer pyrethroid resistance in *An. funestus* was observed in Tororo and Lira mosquitoes populations (2.5< fold change (FC) <3.8) (Figure 6A). These FCs are significantly lower than in Malawi or Mozambique, southern Africa [5]. However, both genes are significantly more over-expressed in Kisumu in Kenya than the two Ugandan locations (P<0.05).

The *GSTe2* gene recently shown to confer DDT resistance in West/Central Africa [27] was also significantly over-expressed in Tororo (FC 3.1) and Lira (FC4.4) in Uganda (P<0.05) but at a significantly much lower rate than the 82-fold change observed in Benin. In addition, the over-expression of *GSTe2* in Kisumu was not significantly different to that of the susceptible FANG strain.

### Role of L119F-GSTe2 mutation in the DDT resistance

Genotyping of the L119F-GSTe2 mutation in Jinja detected the presence of the resistant 119F allele but at a low frequency of 10% (Figure 6B) in contrast to high frequencies observed in West and Central Africa [27]. The frequency of the 119F allele is low even in DDT resistant mosquitoes as 73.3% of resistant were homozygotes for the L119 susceptible allele (Figure 6B). This suggests that a major proportion of the observed DDT resistance in Jinja has no link to the L119F mutation. However, the comparison of the distribution of this mutation between resistant and susceptible mosquitoes revealed that it is nevertheless significantly associated with DDT resistance (odds ratio  = 12.9; P<0.001).

## Discussion

The extent and geographical distribution of insecticide resistance in *An. funestus* populations in Uganda and neighbouring Kenya had remained unknown since the first report of pyrethroid and DDT resistance in the region in 2010 [11]. This study has presented a nationwide distribution of resistance in this major malaria vector across Uganda and has confirmed that this resistance also extends to western Kenya.

Field collections confirmed that *An. funestus s.s* is the predominant species of the *An. funestus* group across Uganda as suggested previously [30], [31] apart from Masindi (central-western Uganda) where both *An. funestus* and *An. parensis* were found in almost equal proportions. The recent report of the predominance of *An. parensis* in western locations of Ntungamo and Mityana (central) [29] and the presence of *An. leesoni* [30] appears to be limited to these region and not nationwide.

### Role in Malaria transmission

The sporozoite infection rates of *An. funestus* populations across Uganda and in the Kisumu population is similar to the rates generally reported for *An. funestus* across the continent [32]. The relatively high infection rates of *An. funestus* in locations such as Lira and Tororo highlights the need of giving further attention to *An. funestus s.s* not just *An. gambiae* when it comes to vector control. The higher infection rates observed in some locations could also be indicative of the presence of hot spots for malaria transmission in Uganda as previously reported in Tanzania [33].

### Characterisation of insecticide resistance profiles

#### Widespread pyrethroid and DDT resistance

This study has revealed that resistance to both type I and II pyrethroids is extensively distributed across Ugandan *An. funestus* populations and also extends to western Kenya as previously suggested [34]. The causes of such widespread resistance remain unknown although use of pyrethroids in agriculture may be playing a role. For example in Kisumu, samples were collected from a village surrounded by a large commercial rice farm - ‘Dominion farm’ where pesticide spraying, including pyrethroids, is routine. A similar situation is observed in Bulambuli with the presence of large cotton farms. Impact of the use of insecticides in the agricultural sector has previously been shown to be a major factor in selecting resistance in malaria vectors [35]. However, a possible role of the scale up of LLINs distribution across Uganda and Kenya cannot be ruled out as a contributing factor [1]. The extensive distribution of pyrethroid resistance in East Africa in addition to what has been reported across southern [3], [4], [6], [36] and West Africa [8] suggests that pyrethroid resistance is now as widespread in *An. funestus* populations across Africa just as in *An. gambiae* [37].

The consistent detection of DDT resistance in all the tested populations also suggests that DDT resistance is widespread in *An. funestus* Ugandan populations and possibly also in East Africa. This is similar to the case of *An. funestus* populations in West and Central Africa [7]–[9] although higher level of resistance was reported in Benin (with no mortality to DDT after 1 h exposure) [8]. In contrast, the extensive distribution of DDT resistance in East Africa is very different from cases of full susceptibility observed in southern Africa notably in Mozambique [4], [38]. The full susceptibility to bendiocarb in most populations tested in this study contrasts to the widespread carbamate resistance in southern Africa [4], [6], [36]. In this respect the East African populations of *An. funestus* also differ to those in West Africa where bendiocarb resistance has been reported [8], [9]. Similar difference is observed for dieldrin resistance which is detected at high levels in West and Central Africa but is absent in East Africa. Overall, the comparison of the susceptibility profile of the Ugandan populations and the western Kenyan population suggests the presence of three distinctive resistance patterns in Africa, one in East with DDT and pyrethroid resistance, one in southern Africa with pyrethroid and carbamates resistance and one in West Africa with resistance to all the three insecticide classes. This difference in resistance profiles possibly reflects the presence of barriers of gene flow between these populations particularly as these three resistance patterns correspond to known genetic clusters defined across Africa [39].

The full susceptibility to malathion confirms that *An. funestus* populations across the continent remain susceptible to this organophosphate insecticide making it a possible alternative insecticide for resistance management strategies.

#### Pyrethroid and DDT resistance is increasing

A comparison of the mortality rate observed for pyrethroids and DDT between the 2009 [11] and the 2012 collection suggests a significant increase of the level of resistance against both pyrethroids and DDT in Tororo. It remains to be confirmed whether this observed increase in the resistance level reflects a true increase of resistance intensity or is just an artefact associated with two different collections possibly impacted by other factors. Indeed it has been shown in *An. gambiae* that resistance level can fluctuate in a same location due among others to seasonal climatic changes [40].

### Pyrethroid and DDT resistance is driven by metabolic resistance not *kdr*

Both pyrethroids and DDT resistance mechanisms are likely not associated with target resistance as no *kdr* mutation associated with both resistance was detected. The F1021C mutation identified in only in Lira was not linked to the DDT resistance as it was only found in two DDT susceptible mosquitoes. However, because this mutation is located in close proximity to the 1014 codon where mutations are commonly associated with both pyrethroid and DDT resistance in other insects [41], this mutation should further be monitored in the field with higher sample size and its potential increase in frequency also assessed. The overall lack of selection observed on the intron 19/Exon 20 VGSC fragment (domain II) across Uganda and in the western Kenya, with high level of polymorphism, further supports that knockdown resistance is probably not playing a significant role in the observed resistance.

Metabolic resistance is likely the major resistance mechanism in these populations notably through elevated cytochrome P450 genes for pyrethroid resistance. This is supported by the near full recovery of susceptibility observed to pyrethroids after PBO exposure and the absence of an association between *kdr* and resistance. Both *CYP6P9a* and *CYP6P9b*, the two main pyrethroid resistance genes in southern Africa [5], were not highly over-expressed in Uganda. A higher expression level for both genes was observed in the Kenyan population from Kisumu but still at a lower level than southern Africa. It is most likely that both genes may play a lesser role in pyrethroid resistance in East Africa than in southern Africa. Similarly, a significantly lower over-expression was observed for the *GSTe2* gene than in West and Central Africa where it is the main DDT resistance gene [27]. This low over-expression correlated well with the low frequency of the resistant 119F-GSTe2 allele in the Jinja population suggesting that unlike West and Central Africa, *GSTe2* plays a lesser role in the DDT resistance observed in East Africa. Nevertheless, the significant correlation observed between L119F genotypes and DDT resistance in this study confirms the ability of the 119F allele to confer DDT resistance in this species. Future genome-wide transcription analysis will help to further elucidate the molecular basis of the pyrethroid and DDT resistance observed in this region.

### Cross-resistance between DDT and pyrethroids is unlikely

The consistent simultaneous resistance to both pyrethroids and DDT in East Africa calls for an investigation whether cross-resistance exists between the two insecticide classes as previously reported in other mosquitoes species such as *An. gambiae* [10], [42], *Aedes aegypti* [43] and *Culex quinquefasciatus* [44]. If such cross resistance exist, it is most likely not operating through knockdown resistance (*kdr*) mutation (as discussed above) contrary to case in *An. gambiae* [42] or *Culex quinquefasciatus* [44]. Such pyrethroids/DDT cross resistance could also operate through metabolic resistance notably via elevated expression of cytochrome P450s as previously reported in *An. gambiae* where the *CYP6M2* gene was demonstrated to confer such cross-resistance [45]. However, the synergist assay with PBO clearly suggests that P450s have little involvement in the DDT resistance observed in East Africa as no significant recovery of susceptibility was observed for DDT after pre-exposure to PBO. This highly contrasted with pyrethroids for which the recovery of susceptibility was high with 90 to 100% susceptibility after PBO pre-exposure. Therefore, a cross-resistance mechanism is unlikely between pyrethroids and DDT and the co-presence of both resistance types most likely results from independent selection factors under different resistance mechanisms.

The impact of the observed pyrethroid resistance on the effectiveness of control interventions, notably LLINs, against *An. funestus* populations in Uganda remains unknown. However, a greater decrease was recently observed in the odds of malaria when switching from DDT or alpha-cypermethrin to carbamates in the Apac district in northern Uganda suggesting that resistance to both pyrethroids and DDT may impact malaria control [46]. In addition, bio-efficacy tests on resistant *An. gambiae* in Uganda [47] has shown that resistance there could lead to reduced efficacy of some LLINs while others such as permanet 3.0 continue to perform well irrespective of resistance.

### Conclusion

The countrywide distribution of pyrethroid and DDT resistance across Ugandan *An. funestus* populations and in the western Kenyan population represents a challenge for the control of this major malaria vector. Assessing the real impact of this pyrethroid resistance on the continued effectiveness of LLINs in both countries should be a priority. However, the full susceptibility to both carbamates and organophosphates currently provide alternative insecticides for the implementation of resistance management strategies such as rotations for IRS. The recent shift to the carbamate bendiocarb for the indoor spraying program across Uganda appears to be a step in the right direction.

## Supporting Information

Figure S1 **Maximum likelihood phylogenetic tree of VGSC fragment with no specific clade associated with resistance to permethrin (A) and to DDT (B).** BL (Balambuli); TR (Tororo); KS (Kisumu); JN (Jinja); Lr (Lira); Pd (permethrin dead mosquito); Pa (Permethrin alive mosquito); Dd (DDT dead mosquito); Da (DDT alive mosquito).
(PDF)

Figure S2 **Maximum likelihood phylogenetic tree of VGSC fragment after combining all resistant and susceptible mosquitoes for both pyrethroid and DDT samples.**
(TIF)

Table S1 **List of primers used.**
(DOCX)

Table S2 **Sporozoite infection rate by location.**
(DOCX)

Table S3 **WHO susceptibility test results following 1 hour exposure to given insecticides.**
(DOCX)
