# Supplementary figures and images for: Widespread Pyrethroid and DDT Resistance in the Major Malaria Vector Anopheles funestus in East Africa Is Driven by Metabolic Resistance Mechanisms
Source: PLoS One. 2014 Oct 15;9(10):e110058. doi: 10.1371/journal.pone.0110058 (PMC4198208; doi:10.1371/journal.pone.0110058)

**A**

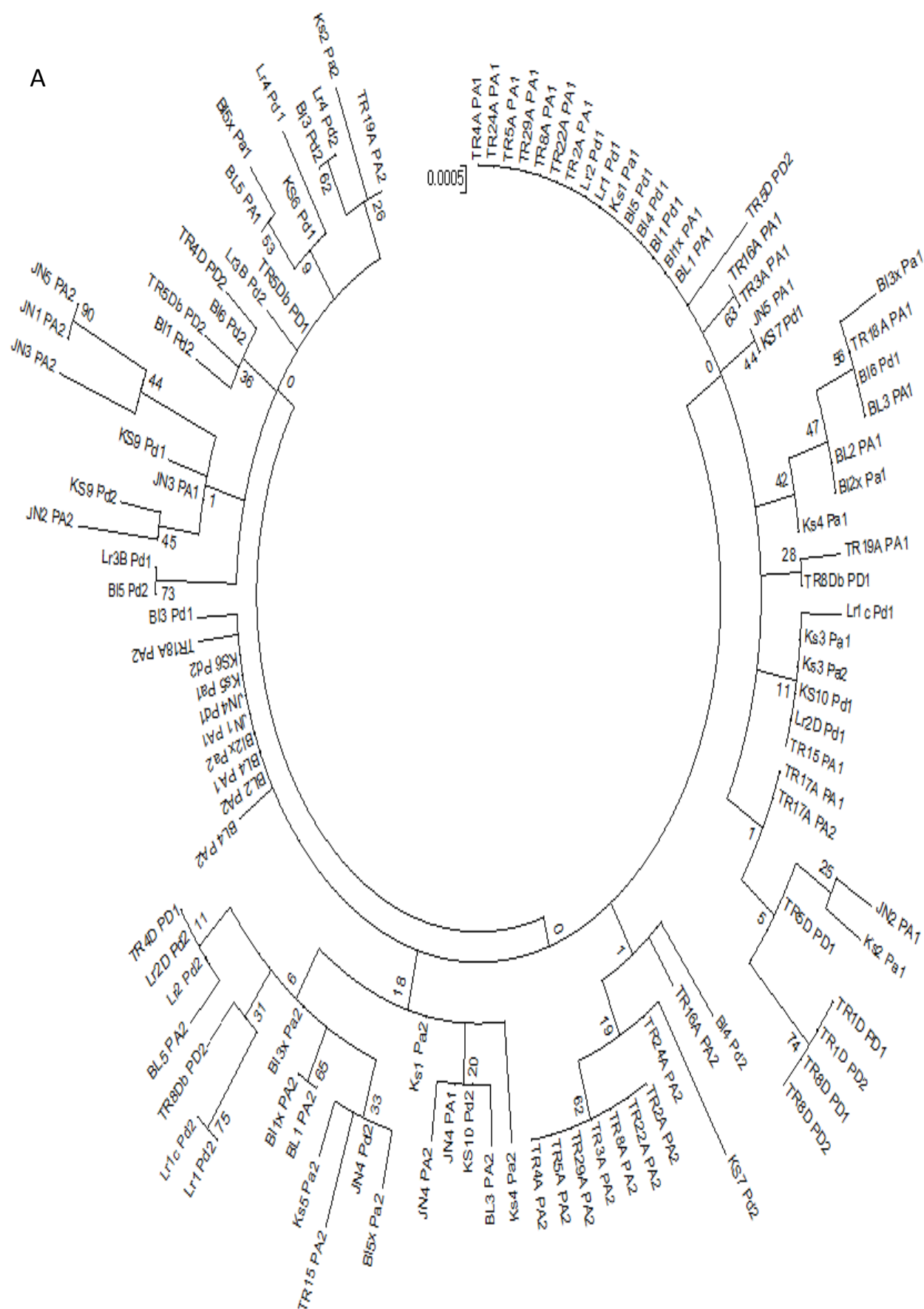

B

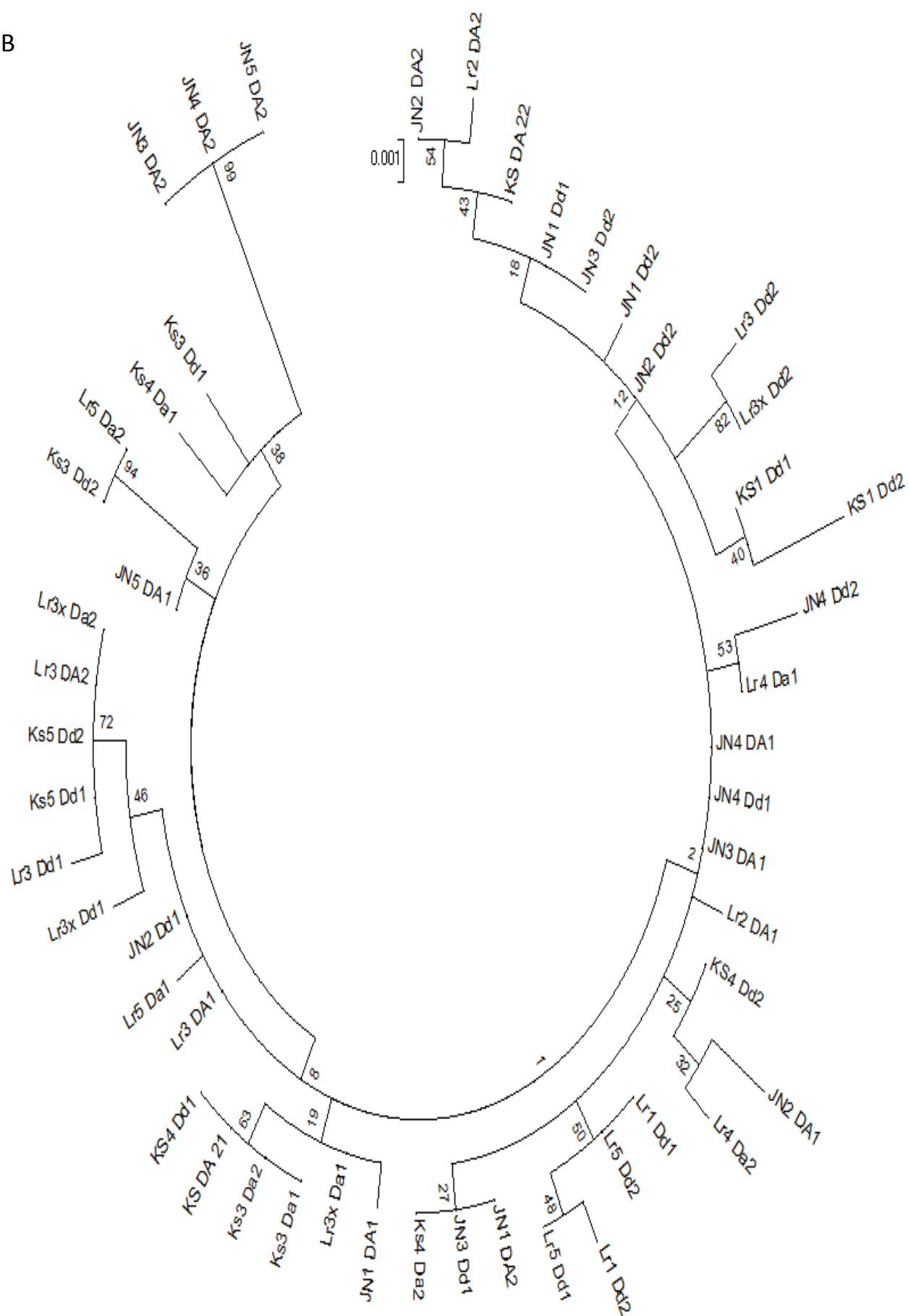

Supplement: Figure S1 — Maximum likelihood phylogenetic tree of VGSC fragment with no specific clade associated with resistance to permethrin (A) and to DDT (B). BL (Balambuli); TR (Tororo); KS (Kisumu); JN (Jinja); Lr (Lira); Pd (permethrin dead mosquito); Pa (Permethrin alive mosquito); Dd (DDT dead mosquito); Da (DDT alive mosquito). (PDF) [file pone.0110058.s001.pdf]

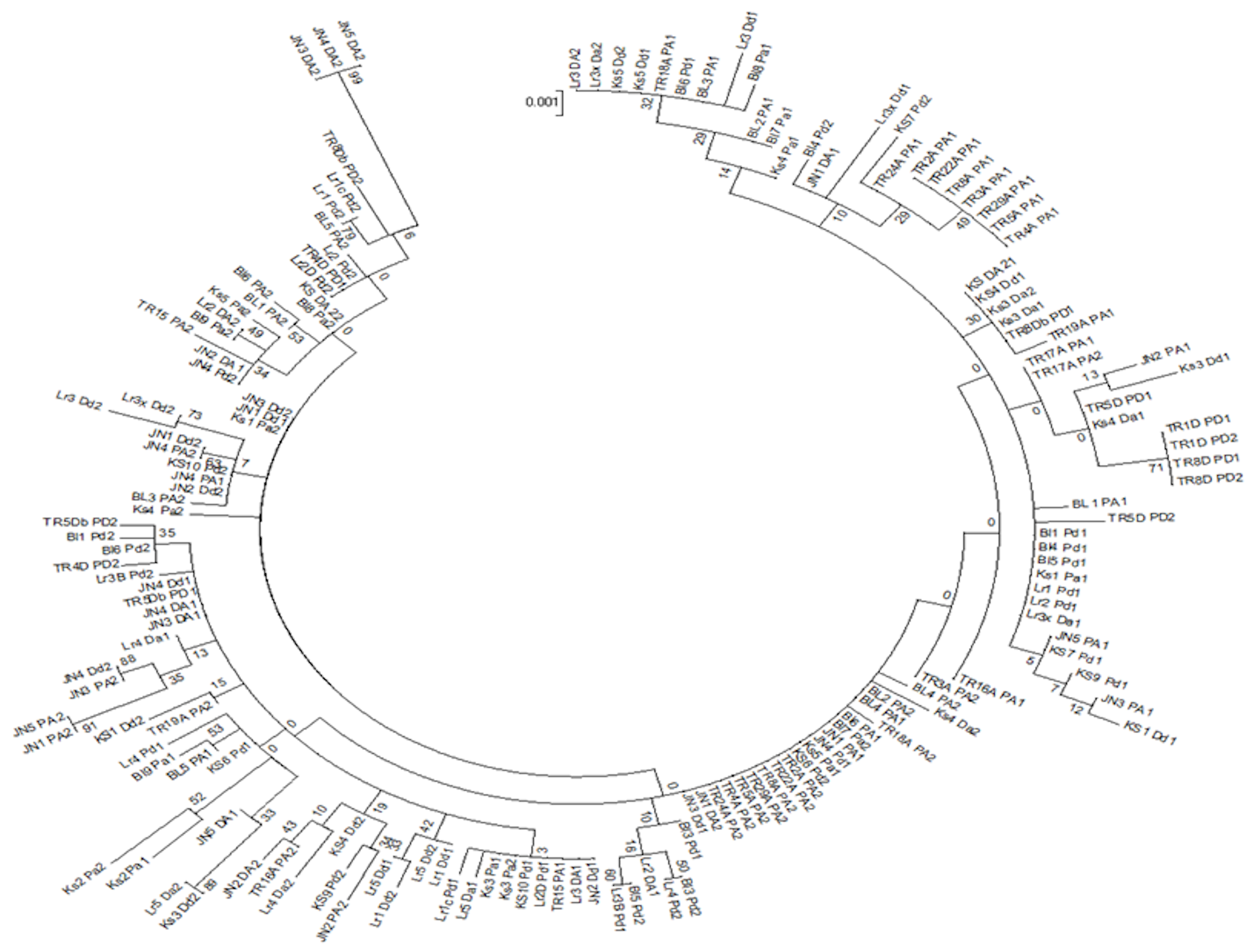

Supplement: Figure S2 — Maximum likelihood phylogenetic tree of VGSC fragment after combining all resistant and susceptible mosquitoes for both pyrethroid and DDT samples. (TIF) [file pone.0110058.s002.tif]
