# Supplementary material for: Widespread Pyrethroid and DDT Resistance in the Major Malaria Vector Anopheles funestus in East Africa Is Driven by Metabolic Resistance Mechanisms
Source: PLoS One. 2014 Oct 15;9(10):e110058. doi: 10.1371/journal.pone.0110058 (PMC4198208; doi:10.1371/journal.pone.0110058)
Supplement: Table S1 — List of primers used. (DOCX) [file pone.0110058.s003.docx]

**Table S1**: List of primers used

| **Primers** | **Forward** | **Reverse** | **Expected size (bp)** | | |
| --- | --- | --- | --- | --- | --- |
| **VGSC** | GTT CAA TGA AGC CCC TCA AA | CCG AAA TTT GAC AAA AGC AAA | | 994 | |
| qRT-PCR | | | | | |
| **CYP6P9a** | CAGCGCGTACACCAGATTGTGTAA | TCACAATTTTTCCACCTTCAAGTAATTACCCGC | | | 92 |
| **CYP6P9b** | CAGCGCGTACACCAGATTGTGTAA | TTACACCTTTTCTACCTTCAAGTAATTACCCGC | | | 97 |
| **CYP6M7** | CCA GAT ACT GAA AGA GAG CCT TCG | CAAGCACTGTCTTCGTACCG | | | 102 |
| **RSP7** | GTGTTCGGTTCCAAGGTGAT | TCCGAGTTCATTTCCAGCTC | | | 98 |
| **ACTIN** | TTAAACCCAAAAGCCAATCG | ACCGGATGCATACAGTGACA | | | 111 |
