# Supplementary material for: Widespread Pyrethroid and DDT Resistance in the Major Malaria Vector Anopheles funestus in East Africa Is Driven by Metabolic Resistance Mechanisms
Source: PLoS One. 2014 Oct 15;9(10):e110058. doi: 10.1371/journal.pone.0110058 (PMC4198208; doi:10.1371/journal.pone.0110058)
Supplement: Table S2 — Sporozoite infection rate by location. (DOCX) [file pone.0110058.s004.docx]

Table S2: Sporozoite infection rate by location

| **Location** | **No. of F0s tested** | **No. of positives** | **% positivity** |
| --- | --- | --- | --- |
|  |  |  |  |
| Lira | 48 | 5 | 10.4 |
| Tororo | 48 | 4 | 8.3 |
| Bulambuli | 49 | 3 | 6.1 |
| Kisumu | 45 | 2 | 4.4 |
| Masindi | 48 | 2 | 4.2 |
| Arua | 48 | 2 | 4.2 |
