# Supplementary material for: Widespread Pyrethroid and DDT Resistance in the Major Malaria Vector Anopheles funestus in East Africa Is Driven by Metabolic Resistance Mechanisms
Source: PLoS One. 2014 Oct 15;9(10):e110058. doi: 10.1371/journal.pone.0110058 (PMC4198208; doi:10.1371/journal.pone.0110058)
Supplement: Table S3 — WHO susceptibility test results following 1 hour exposure to given insecticides. (DOCX) [file pone.0110058.s005.docx]

**Table S3**: WHO susceptibility test results following 1hour exposure to given insecticides

|  |  | **Arua (Ar)** | | **Bulambuli (Bl)** | | **Jinja (Jn)** | | **Lira (Lr)** | | **Tororo (Tr)** | | **Kisumu (Ks)** | | **Masindi (Ms)** | |
| --- | --- | --- | --- | --- | --- | --- | --- | --- | --- | --- | --- | --- | --- | --- | --- |
|  |  | **n** | **% mortality** | **n** | **% mortality** | **n** | **% mortality** | **n** | **% mortality** | **n** | **% mortality** | **n** | **% mortality** | **n** | **% mortality** |
| Permethrin | F | 96 | 27±2.7 | 93 | 49±9.7 | 98 | 6±1.2 | 80 | 51±9.4 | 96 | 33±2.7 | 92 | 20±6.8 | 93 | 73±4.3 |
|  | M | 95 | 47±13.2 | 93 | 66±9.7 | 97 | 33±11.9 | 79 | 66±7.6 | 92 | 62±8.2 | 94 | 23±1.4 | 83 | 82±2.1 |
| Deltamethrin | F | 96 | 20±1.6 | 98 | 29±4.3 | 100 | 4±1.6 | 75 | 4±2.3 | 95 | 20±4.4 | 95 | 8±4.3 | 75 | 53±2.5 |
|  | M | 97 | 29±1.9 | 91 | 65±7.9 | 96 | 9±0.9 | 80 | 7±3.5 | 95 | 17±3.4 | 95 | 21±8.9 | 70 | 72±6.1 |
| DDT | F | 91 | 40±7.8 | 96 | 64±4.2 | 100 | 42±6.2 | 68 | 48±4.0 | 95 | 61±4.0 | 95 | 52±3.3 | 90 | 69±2.3 |
|  | M | 86 | 64±4.3 | 91 | 75±7.4 | 96 | 48±7.3 | 68 | 81±1.7 | 95 | 80±1.8 | 92 | 79±5.0 | 70 | 93±2.5 |
| Lambda-cyhalothrin | F | / | – | 72 | 8±2.4 | / | – | / | – | 50 | 22±2 | / | – | / | – |
|  | M | / | – | 75 | 33±5.9 | / | – | / | – | 50 | 10±2 | / | – | / | – |
| Etofenprox | F | / | – | 73 | 66±5.7 | / | – | / | – | 25 | 20±0 | / | – | / | – |
|  | M | / | – | 77 | 76±2.3 | / | – | / | – | 21 | 23±0 | / | – | / | – |
| Bendiocarb | F | / | – | 73 | 98±2.0 | 49 | 100±0 | 56 | 100±0 | 25 | 96±0 | / | – | / | – |
|  | M | / | – | 73 | 100±0 | 51 | 100±0 | 60 | 100±0 | 25 | 100±0 | / | – | / | – |
| Malathion | F | / | – | 45 | 98±2.0 | 50 | 100±0 | 48 | 100±0 | 25 | 100±0 | / | – | / | – |
|  | M | / | – | 45 | 100±0 | 52 | 100±0 | 45 | 100±0 | 25 | 100±0 | / | – | / | – |
| Dieldrin | F | / | _ | 20 | 100± | / | _ | / | _ | / | _ | / | _ | / | _ |
|  | M | / | _ | 25 | 100± | / | _ | / | _ | / | _ | / | _ | / | _ |
| Fenitrothion | F | / | _ | 25 | 100± | / | _ | / | _ | / | _ | / | _ | / | _ |
|  | M | / | _ | 25 | 100± | / | _ | / | _ | / | _ | / | _ | / | _ |
| **Total** | **F** | **283** |  | **595** |  | **397** |  | **327** |  | **411** |  | **282** |  | **258** |  |
|  | **M** | **278** |  | **595** |  | **392** |  | **332** |  | **403** |  | **281** |  | **223** |  |
